# Supplementary material for: Monitored long-range interacting systems: spin-wave theory for quantum trajectories
Source: Nat Commun. 2025 May 9;16:4329. doi: 10.1038/s41467-025-59557-w (PMC12064665; doi:10.1038/s41467-025-59557-w)
Supplement: Supplementary file 1 — Supplementary Information [file 41467_2025_59557_MOESM1_ESM.pdf]

Supplementary Information for *Monitored long-range interacting systems: spin-wave theory for quantum trajectories*

Zejian Li,[1] Anna Delmonte,[2] Xhek Turkeshi,[3] and Rosario Fazio[1,4]
[1] *The Abdus Salam International Center for Theoretical Physics, Strada Costiera 11, 34151 Trieste, Italy*
[2] *SISSA, Via Bonomea 265, I-34136 Trieste, Italy*
[3] *Institut für Theoretische Physik, Universität zu Köln, Zülpicher Strasse 77, 50937 Köln, Germany*
[4] *Dipartimento di Fisica "E. Pancini", Università di Napoli "Federico II", Monte S. Angelo, I-80126 Napoli, Italy*

SUPPLEMENTARY NOTE I: PROOF OF THE UNRAVELING OF THE NONDIAGONAL LINDBLAD MASTER EQUATION

In the nondiagonal master equation (6) of the main text, the positive semidefinite matrix f_{ij} can be diagonalized with a unitary transformation u:

$$f = u\kappa u^\dagger \,, \tag{S1}$$

where κ is a diagonal matrix with non-negative entries:

$$\kappa \equiv \mathrm{diag}(\kappa_1, \ldots, \kappa_N)\,. \tag{S2}$$

Defining

$$\begin{aligned} \hat{A}_i &\equiv \sum_j u_{ji}\hat{L}_j \,, \\ \mathrm{d}Z_i &\equiv \frac{1}{\sqrt{\kappa_i}} \sum_j u_{ji}\mathrm{dw}_j \,, \end{aligned} \tag{S3}$$

the Liouvillian (6) in the main text becomes

$$\mathcal{L}(\hat\rho) = -\mathrm{i}[\hat{H}, \hat\rho] + \sum_i \kappa_i \mathcal{D}[\hat{A}_i](\hat\rho)\,, \tag{S4}$$

where

$$\mathcal{D}[\hat{A}_i](\hat\rho) \equiv \hat{A}_i\hat\rho\hat{A}_i^\dagger - \frac{1}{2}\left\{\hat{A}_i^\dagger\hat{A}_i, \hat\rho\right\} \tag{S5}$$

is the standard (diagonal) dissipator. The unraveling (1) in the main text becomes

$$\begin{aligned} \mathrm{d}\hat\rho &= \mathrm{d}t\mathcal{L}(\hat\rho) \\ &+ \sum_i \sqrt{\kappa_i}\left[\mathrm{d}Z_i^*\left(\hat{A}_j - \langle\hat{A}_i\rangle\right)\hat\rho + \mathrm{d}Z_i\hat\rho\left(\hat{A}_i^\dagger - \langle\hat{A}_i^\dagger\rangle\right)\right]\,, \end{aligned} \tag{S6}$$

where the noise satisfies

$$\begin{aligned} &\overline{\mathrm{d}Z_i} = 0\,, \\ &\mathrm{d}Z_i^*\mathrm{d}Z_j = \delta_{ij}\mathrm{d}t\,, \quad \mathrm{d}Z_i\mathrm{d}Z_j = 0\,, \end{aligned} \tag{S7}$$

which implies that each $\mathrm{d}Z_i$ is a normalized complex Wiener process and independent from each other. Eq. (S6), being equivalent to Eq. (1) of the main text, is the standard (diagonal-form) quantum state diffusion unraveling describing a heterodyne detection process, where the operators $\hat{A}_i$ are being continuously monitored at rates κ_i respectively.

For a pure initial state, this is equivalent to the following stochastic Schrödinger equation,

$$\begin{aligned} \mathrm{d}|\psi\rangle &= -\mathrm{i}\hat{H}\mathrm{d}t|\psi\rangle \\ &+ \sum_i \kappa_i\left(\left\langle\hat{A}_i^\dagger\right\rangle\hat{A}_i - \frac{1}{2}\left\langle\hat{A}_i^\dagger\right\rangle\left\langle\hat{A}_i\right\rangle - \frac{1}{2}\hat{A}_i^\dagger\hat{A}_i\right)\mathrm{d}t|\psi\rangle \\ &+ \sum_i \sqrt{\kappa_i}\left(\hat{A}_i - \left\langle\hat{A}_i\right\rangle\right)\mathrm{d}Z_i^*|\psi\rangle\,, \end{aligned} \tag{S8}$$

which preserves the purity of the state along every single trajectory.

SUPPLEMENTARY NOTE II: MEAN-FIELD EQUATIONS FOR THE POWER-LAW SPIN MODEL

We derive in this Section the mean-field equations of the power-law spin model defined in the main text. We denote the average magnetization with

$$m_\mu \equiv \frac{\langle\hat{S}^\mu\rangle}{S} = \frac{1}{N}\sum_{i=1}^N \langle\hat\sigma_i^\mu\rangle\,, \quad \mu\in\{x,y,z\}\,, \tag{S9}$$

whose evolution under the Lindblad dynamics can be obtained with the adjoint master equation [1]: for a time-independent operator $\hat{O}$, we have

$$\frac{\mathrm{d}\langle\hat{O}\rangle}{\mathrm{d}t} = \mathrm{i}\left\langle[\hat{H},\hat{O}]\right\rangle + \frac{\kappa}{S}\left\langle\hat{S}^+\hat{O}\hat{S}^- - \frac{1}{2}\left\{\hat{S}^+\hat{S}^-, \hat{O}\right\}\right\rangle. \tag{S10}$$

Within the mean-field approximation, where we assume the factorization $\langle\hat\sigma_i^\mu\hat\sigma_j^\lambda\rangle = \langle\hat\sigma_i^\mu\rangle\langle\hat\sigma_j^\lambda\rangle$ at $N\to\infty$, and that $\langle\hat\sigma_i^\mu\rangle$ has no $i-$dependence, we obtain the following equations of motion for the average magnetization:

$$\begin{aligned} \frac{\mathrm{d}m_x}{\mathrm{d}t} &= -4Jm_ym_z + \kappa m_xm_z\,, \\ \frac{\mathrm{d}m_y}{\mathrm{d}t} &= -\omega m_z + 4Jm_xm_z + \kappa m_ym_z\,, \\ \frac{\mathrm{d}m_z}{\mathrm{d}t} &= \omega m_y - \kappa(m_x^2 + m_y^2)\,. \end{aligned} \tag{S11}$$

The steady-state magnetization can be found by imposing the time derivatives to zero, which yields

$$m_z = -\sqrt{1 - \frac{\omega^2}{16J^2+\kappa^2}}\,. \tag{S12}$$

Beyond the critical point $\omega_{\rm MF}^{(c)} = \sqrt{16J^2+\kappa^2}$, this stationary solution no longer exists and the magnetization admits only permanently oscillating solutions with zero mean when averaged over long times. The mean-field theory therefore predicts a continuous phase transition from a stationary phase to a time-crystal phase on the level of the Lindblad dynamics of the average state.

SUPPLEMENTARY NOTE III: EXPRESSION FOR THE ENTANGLEMENT ENTROPY UNDER THE GAUSSIAN APPROXIMATION

Without loss of generality, let us consider a bipartition of the spins where one subsystem contains spins indexed from 1 to M. Denoting $\hat{x}_i \equiv (\hat{b}_i^\dagger + \hat{b}_i)/\sqrt{2}$, $\hat{p}_i \equiv \mathrm{i}(\hat{b}_i^\dagger - \hat{b}_i)/\sqrt{2}$ and $\hat{\mathbf{r}} \equiv (\hat{x}_1, \ldots, \hat{x}_M, \hat{p}_1, \ldots, \hat{p}_M)$, the covariance matrix $\mathbf{\Xi}$ for the Gaussian state of the subsystem has components

$$\Xi_{ij} \equiv \frac{1}{2}\langle \hat{r}_i\hat{r}_j + \hat{r}_j\hat{r}_i\rangle - \langle \hat{r}_i\rangle\langle \hat{r}_j\rangle\,, \tag{S13}$$

which can be expressed in terms of u_{ij} and v_{ij}. The entanglement entropy S_E is then given by the von Neumann entropy of the subsystem (since the full system remains in a pure state along the unraveled trajectory), which can be expressed in terms of the symplectic eigenvalues $\{\mu_i\}_i$ of the covariance matrix $\mathbf{\Xi}$ as [2]

$$\begin{aligned} S_E = \sum_i \bigg[&\left(\mu_i + \frac{1}{2}\right)\log\left(\mu_i + \frac{1}{2}\right) \\ &-\left(\mu_i - \frac{1}{2}\right)\log\left(\mu_i - \frac{1}{2}\right)\bigg]\,. \end{aligned} \tag{S14}$$

Operationally, the symplectic eigenvalues can be found with the help of the symplectic matrix $\mathbf{\Omega}$ defined as

$$\mathbf{\Omega} = \begin{pmatrix} \mathbf{0} & \mathbf{I}_M \\ -\mathbf{I}_M & \mathbf{0} \end{pmatrix}, \tag{S15}$$

where $\mathbf{I}_M$ is the $M\times M$ identity matrix. The eigenvalues of the matrix $\mathbf{\Xi\Omega}$ are then $\{\pm \mathrm{i}\mu_i\}_i$, where the μ_i's are the symplectic eigenvalues of $\mathbf{\Xi}$.

SUPPLEMENTARY NOTE IV: ADDITIONAL NUMERICAL RESULTS

A Benchmark of SWQT on the steady-state of the infinite-range model

In Supplementary Fig. 1, we compare the steady-state of trajectory average quantities from the spin-wave solutions on the infinite-range model as considered in the main text, against the exact ones, for a wide range of the drive ω. Panel (a) shows the magnetization and panel (b) shows the half-system entanglement entropy. Panel (c) shows the spin-wave density $\bar{\epsilon}$ associated with the spin-wave solutions. The peak in $\bar{\epsilon}$ corresponds to the maximum in the entanglement entropy [in panel (b)], where the difference between the spin-wave and exact solutions is also more pronounced. This illustrates the significance of the control parameter $\bar{\epsilon}$ as a signal for the validity of the spin-wave theory. On the other hand, the qualitative behavior of the entanglement is correctly captured despite the relatively high spin-wave density at its peak, and the magnetization predicted by the spin-wave method [panel (a)] remains accurate for all values of ω considered.

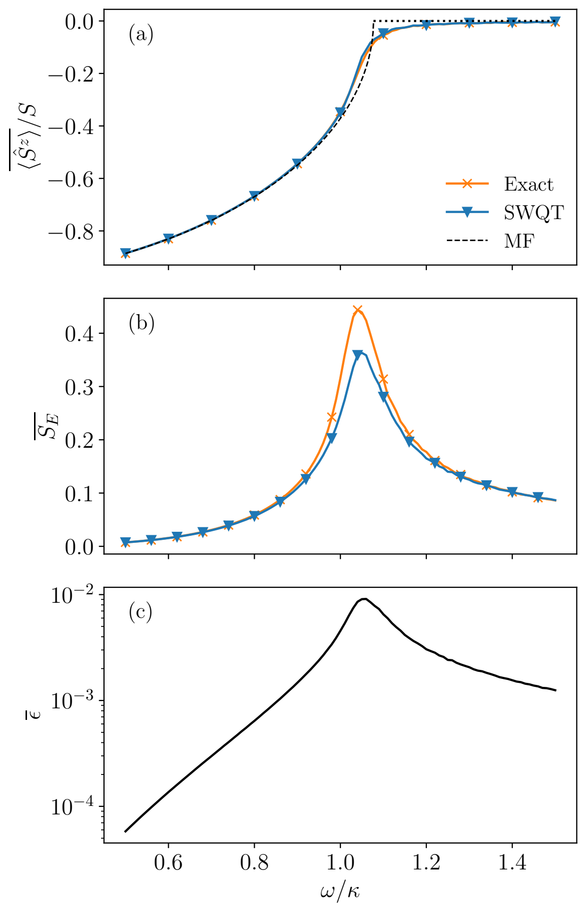

Supplementary Figure 1. Benchmark of the spin-wave method on the steady state of the collective model with $S = 64$ and $J = 0.1\kappa$. (a) Expectation of the collective z-magnetization as a function of the drive ω obtained with the spin-wave quantum trajectories and the exact solution (see legend). The mean-field solution (see supplementary) is marked with the dashed line. (b) Trajectory-averaged steady-state half-chain entanglement entropy obtained with the two methods [see legend in (a)]. (c) Trajectory-averaged spin-wave density $\bar{\epsilon}$ in the steady state.

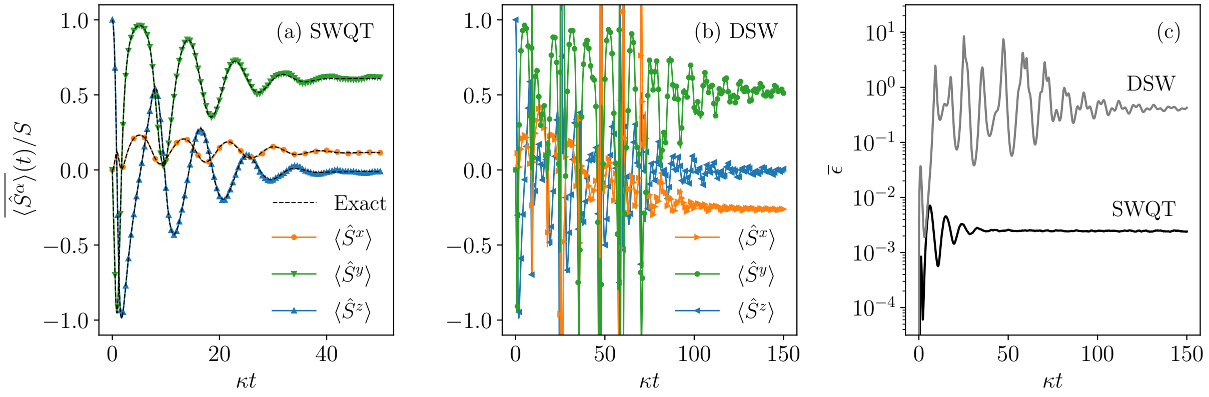

Supplementary Figure 2. Comparison between solutions given by the two different spin-wave methods. (a) Results from the spin-wave quantum trajectories (SWQT) on the expectation value of the collective spin vector in the time-crystal regime with parameters $\omega_F = 1.25\kappa$, $J = 0.1\kappa$ and $S = 64$. (b) The solution given by the deterministic spin-wave theory (DSW) with the same parameters. (c) Spin-wave densities associated with the two solutions.

B Comparison with other existing spin-wave methods

In this section, we provide a comparison between our method of spin-wave quantum trajectories (SWQT) and the method proposed in [3], which is a deterministic spin-wave method for dissipative systems (referred to as "DSW" in this section). The latter assumes the same approximations as in our method (i.e. lowest-order Holstein-Primakoff expansion and Gaussian approximation) except that their approximations are performed on the level of the averaged state (i.e. the density matrix) and their dynamical evolution is derived from the deterministic Lindblad master equation.

For illustration purposes, let us consider again the collective spin model defined in the main text. The solutions given by the two methods are shown in Supplementary Fig. 2 for comparison. Panel (a) shows the SWQT results for the parameters $\omega_F = 1.25\kappa$, $J = 0.1\kappa$, $S = 64$. The solution with the same parameters given by DSW is shown in panel (b), which is by no means close to the exact solution. As explained in the main text, this is a result of the highly mixed nature of the average state that the spin-wave approximations fail to capture. Panel (c) shows the spin-wave densities associated with the two solutions, where the SWQT has a spin-wave density that is smaller by several orders of magnitude, which is consistent with its accuracy compared to the exact solution.

C Additional results for the power-law spin model with $\alpha = 0.2$ in 1D

We study the scaling of the steady-state spin-wave density of the power-law spin model on a 1D chain with $\alpha = 0.2$ as considered in the main text, as shown in Supplementary Fig. 3 (a). When the system size N increases, the spin-wave density is suppressed (including its peak value), which is a signature of the long-range nature of the model: in the $N \to \infty$ limit, a vanishing spin-wave density suggests that the system becomes equivalent to a mean-field (infinite-range) one. In Supplementary Fig. 3 (b), we study the time evolution of the trajectory-averaged entanglement entropy at a driving value $\omega = 1.06\kappa$, which is close to where the maximum steady-state entanglement is achieved for the finite system sizes we studied. Note that we are increasing N exponentially between the different considered values, while the entanglement quickly reaches the steady-state value in all cases, suggesting the mitigation of the exponential overhead for observing the entanglement transition via brute-force post selection.

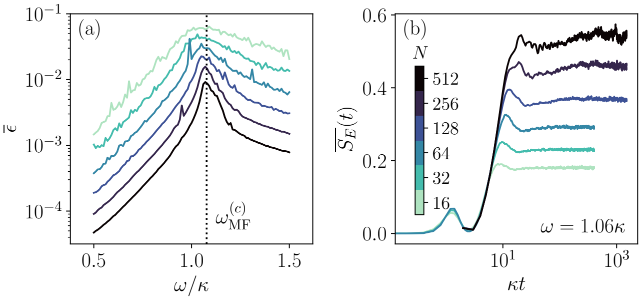

Supplementary Figure 3. Results for the power-law interacting model in the case of $\alpha = 0.2$, $J = 0.1\kappa$ of different system sizes in 1D (colorbar shared across panels). (a) The scaling of the steady-state average spin-wave density $\overline{\epsilon}$ as a function of the drive ω, in log-linear scale. The vertical dotted lines mark the critical point predicted by the mean-field theory $\omega^{(c)}_{\mathrm{MF}} \simeq 1.077\kappa$. (b) Time evolution of the trajectory-average entanglement entropy at a driving value close to criticality $\omega = 1.06\kappa$, in linear-log scale.

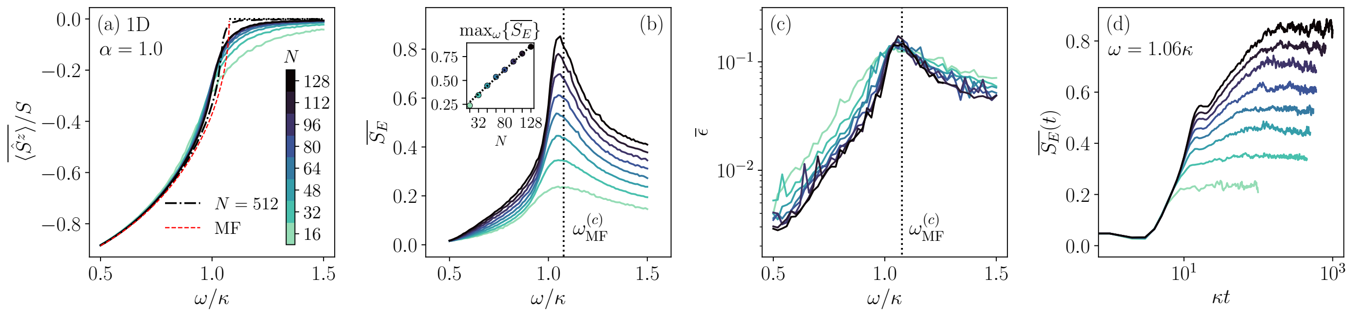

Supplementary Figure 4. Results for the power-law interacting model in the case of $\alpha = 1.0$, $J = 0.1\kappa$ for different system sizes in 1D (colorbar shared across all panels). (a) Steady-state average z magnetization as a function of the drive ω. The dashed line marks the mean-field solution. (b) Steady-state of the trajectory-averaged half-chain entanglement entropy as a function of ω. The vertical dotted lines mark the critical point predicted by the mean-field theory $\omega_{\mathrm{MF}}^{(c)} \simeq 1.077\kappa$. Inset: scaling of the maximum entropy versus N in linear scale. The dotted line is a linear fit for N ranging from 32 to 128 and we report the coefficient of determination to be $R^2 = 0.9980$. (c) Steady-state spin-wave density in log-linear scale. (d) Dynamics of the trajectory-averaged half-chain entanglement entropy for a driving value $\omega = 1.06\kappa$, in linear-log scale.

D Results for the power-law spin model with $\alpha = 1$ in 1D

To study the effect of the interaction range on the entanglement dynamics in the power-law spin model, we also consider the shorter-range case with $\alpha = 1$ on a 1D chain for comparison. The steady-state magnetization $\overline{\langle \hat{S}^z \rangle}$, as shown in Supplementary Fig. 4 (a), exhibits qualitatively similar behavior to the long-range case, while it shows more deviation from the mean-field prediction. This could result from more significant finite-size effects due to short-range interactions. (Note that the mean-field theory does not account for the interaction range parameter α and therefore gives identical predictions regardless of α.) In sharp contrast, Supplementary Fig. 4 (b) shows that the entanglement entropy of the short-range systems grows much faster with the system size N. Finite-size scaling suggests a volume law for the maximum entanglement entropy as well as in a vicinity around the critical point. The spin-wave density, as shown in Supplementary Fig. 4 (c), also presents a qualitatively different behavior from the long-range case. Close to the maximal entanglement, the spin-wave density does not appear to decrease with the system size N, and remains around $\overline{\epsilon} \lesssim 0.2$. This suggests that the short-range model does not reduce to a mean-field (infinite-range) one even in the thermodynamic limit of $N \to \infty$, and that higher-order corrections to the theory are expected to have a more significant contribution. Finally, we show in Supplementary Fig. 4 (d) the time dynamics of the trajectory-averaged entanglement entropy at $\omega = 1.06\kappa$. Contrary to the fast saturation of entanglement in the long-range (or infinite-range) regime, the time it takes to reach the steady-state value scales rapidly with N. Despite the relatively high spin-wave density at the considered driving, we expect our result to hold qualitatively.

E Results for the power-law spin model in 2D

In this section, we present additional results on the power-law spin model for a 2D square lattice. Supplementary Fig. 5 shows the steady-state magnetization [panel (a)], trajectory-averaged half-system entanglement entropy [panel (b)], and the spin-wave density [panel (c)], for the case of $\alpha = 0.2$. As the system size $N = L^2$ increases, the magnetization shows the (dissipative) phase transition predicted by the mean-field theory. The entanglement also develops a log divergence at the same critical point, accompanied by a maximal spin-wave density (with respect to ω) that decreases with N. As the interaction range is sufficiently long, these results are similar to the 1D case with $\alpha = 0.2$ discussed in the main text, and both instances are showing essentially the mean-field (infinite-range) physics. Note that we report results for up to $N = 32 \times 32 = 1024$ interacting spins in 2D.

Supplementary Fig. 6 shows the results for $\alpha = 2.0$, which is the boundary case between our identification of long-range and short-range regimes. Similar to the 1D case with $\alpha = 1.0$, the entanglement scaling at its maximum becomes asymptotically volume-law. The peak spin-wave density no longer vanishes with N, yet still remains bounded and small (< 0.1), suggesting the validity of the spin-wave approximations. The transition in $\langle \hat{S}^z \rangle$ remains qualitatively similar to the mean-field solution (since this transition is driven by the infinite-range dissipation and not the coherent interaction term), with a small deviation originating from the quantum fluctuations close to the transition. These features are more manifest in Supplementary Fig. 7, where we study a moderately short-range case with $\alpha = 3.0$. The entanglement exhibits a volume-law scaling again and the magnetization shows more deviation from the mean-field solution due to the increased quantum fluctuations. Contrary to the previous cases, the spin-wave density increases with

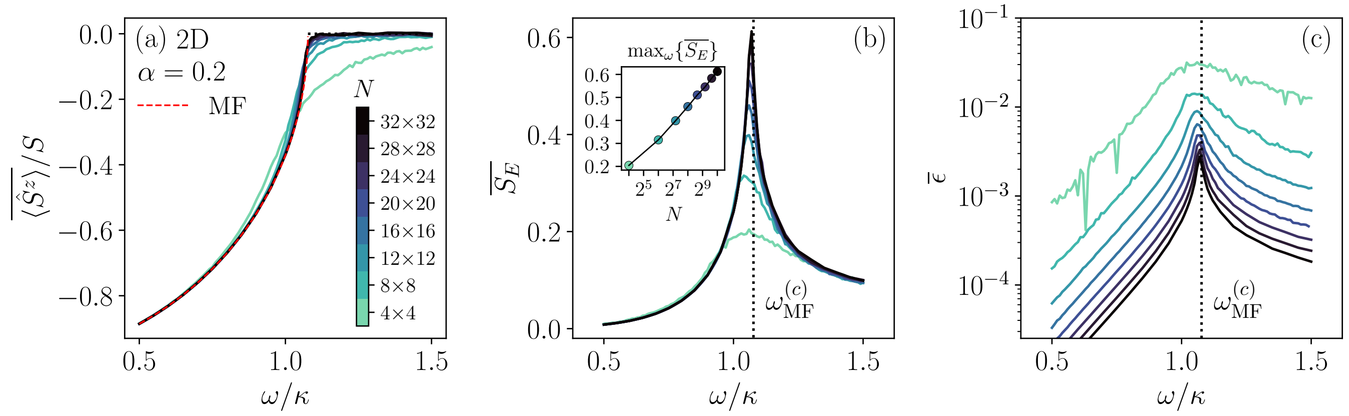

Supplementary Figure 5. Results for the power-law spin model in 2D with $\alpha = 0.2$, $J = 0.1\kappa$ for the following trajectory-averaged quantities in the steady state: (a) z magnetization $\overline{\langle \hat{S}^z \rangle}/S$ with the mean-field solution marked by the dashed line, (b) half-system entanglement entropy $\overline{S_E}$ with its maximum value in the inset, and (c) spin-wave density $\overline{\epsilon}$, as functions of the drive ω for different system sizes $N = L^2$ (see colorbar).

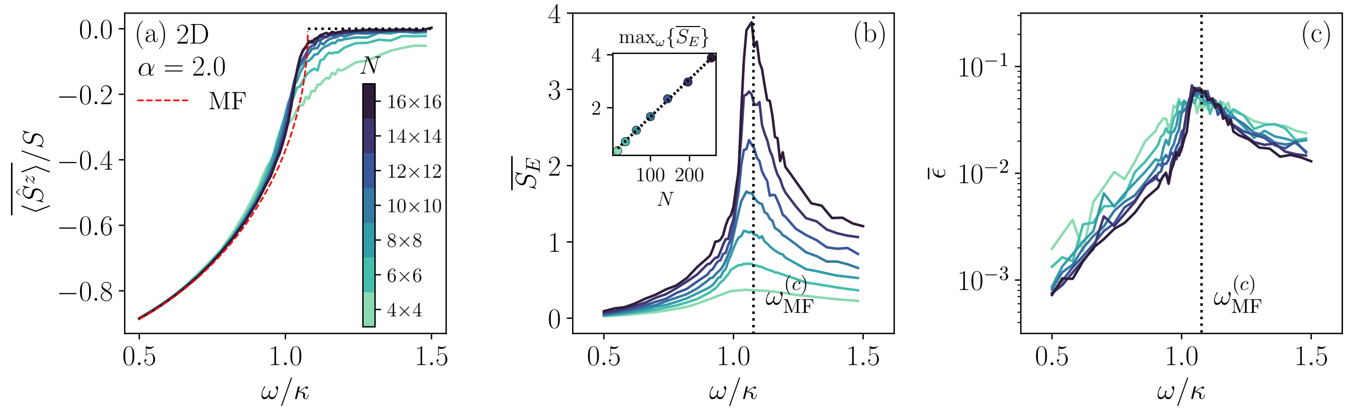

Supplementary Figure 6. Same as Supplementary Fig. 5 but for $\alpha = 2.0$: (a) z magnetization $\overline{\langle \hat{S}^z \rangle}/S$ with the mean-field solution marked by the dashed line, (b) half-system entanglement entropy $\overline{S_E}$ with its maximum value in the inset, and (c) spin-wave density $\overline{\epsilon}$, as functions of the drive ω for different system sizes $N = L^2$ (see colorbar). The dotted line in the inset of panel (b) is a linear fit for N ranging from 36 to 256 and we report the coefficient of determination to be $R^2 = 0.9991$.

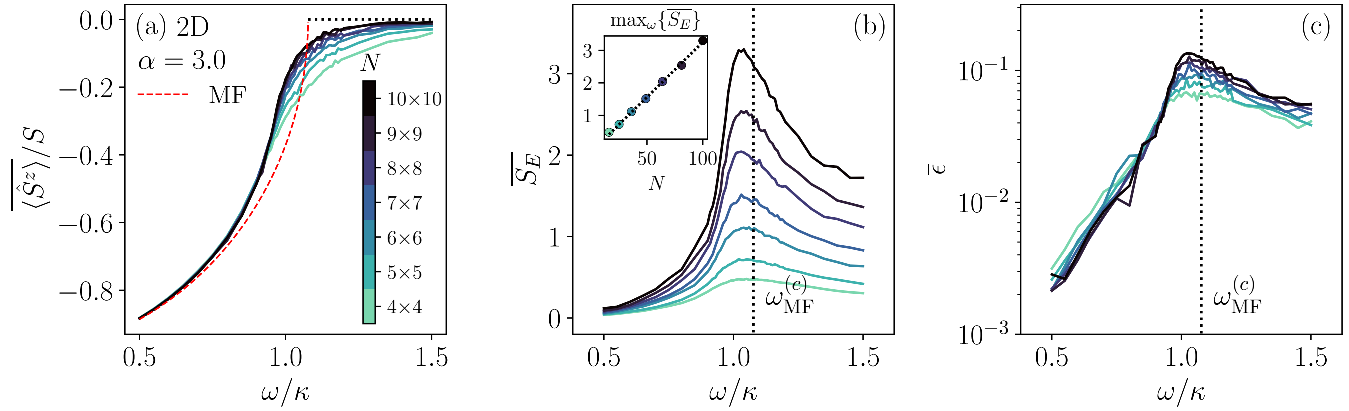

Supplementary Figure 7. Same as Supplementary Fig. 5 but for $\alpha = 3.0$: (a) z magnetization $\overline{\langle \hat{S}^z \rangle}/S$ with the mean-field solution marked by the dashed line, (b) half-system entanglement entropy $\overline{S_E}$ with its maximum value in the inset, and (c) spin-wave density $\overline{\epsilon}$, as functions of the drive ω for different system sizes $N = L^2$ (see colorbar). The dotted line in the inset of panel (b) is a linear fit for N ranging from 25 to 100 and we report the coefficient of determination to be $R^2 = 0.9983$.

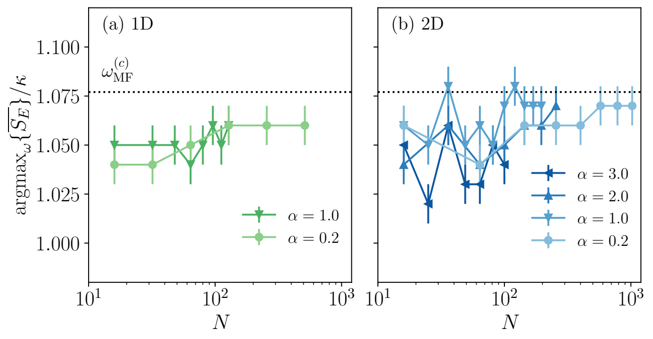

Supplementary Figure 8. The drive that maximizes the steady-state trajectory-averaged entanglement entropy, $\mathrm{argmax}_\omega\{\overline{S_E}\}$, as a function of the system size N for different interaction ranges α respectively in the (a) 1D and (b) 2D model. The errorbar is set at the increment at which we scan ω, i.e. $\Delta\omega = 0.01\kappa$ in our settings. The dotted horizontal line marks the mean-field critical drive value $\omega_{\mathrm{MF}}^{(c)}$.

the system size N for drive values close to and beyond the mean-field critical point. We therefore limit the system sizes studied (up to $N = 10 \times 10 = 100$ spins in 2D) such that the spin-wave density remains small around $\bar{\epsilon} \lesssim 0.1$.

Finally, we show in Supplementary Fig. 8 the drive value at which maximum entanglement is achieved, i.e. $\mathrm{argmax}_\omega\{\overline{S_E}\}$, for the different cases we considered in both 1D and 2D. As the system size N increases, this drive value becomes asymptotically independent of N and converges to a value close to the mean-field critical point.

F Benchmark of the binary approximation of the Gaussian noise

In this section, we verify the validity of the binary approximation of the Gaussian noise presented in the Methods section by computing the trajectory-averaged quantities including both linear and non-linear functions of the state, and by comparing the results with the exact solution obtained with the Gaussian noise. An example of the benchmark is shown in Supplementary Fig. 9, where we computed the magnetization $\overline{\langle \hat{S}^z \rangle}$, the half-system entanglement entropy $\overline{S_E}$ and the nonlinear quantity $\overline{\langle \hat{S}^z \rangle^2}$ from three different methods: 1) Spin-wave quantum trajectories with binarized noise, 2) exact solution of the stochastic master equation with Gaussian noise and 3) exact solution with the binary approximation of the noise. In all cases, we observe excellent agreement among the results obtained in these different ways.

SUPPLEMENTARY NOTE V: GENERALIZATION TO SPIN-BOSON SYSTEMS

In this Section, we demonstrate a possible extension of our spin-wave theory, which allows the investigation of spin-boson systems. We adopt the same Gaussian approximation for the bosonic mode as for the bosonized spins, such that the state of the entire system can be characterized by the first and second moments, requiring a quadratic number of variables to specify the Gaussian variational ansatz in the general case. For illustration purposes, let us consider a driven-dissipative Tavis-Cummings model with long-range spin interactions. The spins are collectively driven and resonantly coupled to a single-mode cavity (with annihilation operator $\hat{a}$ for the cavity bosonic mode), and the cavity undergoes single-photon dissipation. The Hamiltonian can be written as follows,

$$\begin{aligned} \hat{H} = \omega \hat{S}^x + \frac{2sJ}{\mathcal{N}} \sum_{i \neq j} \frac{\hat{\sigma}_i^z \hat{\sigma}_j^z}{\|\mathbf{r}_i - \mathbf{r}_j\|^\alpha} \\ + \frac{\lambda}{\sqrt{2S}} (\hat{a}^\dagger \hat{S}^- + \hat{a} \hat{S}^+) \,, \end{aligned} \tag{S16}$$

which is essentially the spin Hamiltonian (7) in the main text with an additional term (in the second line) describing the spin-boson coupling (with strength λ). The average dynamics of the system can be described by the Lindblad master equation,

$$\frac{\mathrm{d}}{\mathrm{d}t}\hat{\rho} = -\mathrm{i}[\hat{H}, \hat{\rho}] + \kappa \mathcal{D}[\hat{a}](\hat{\rho}) \,, \tag{S17}$$

where κ represents the loss rate of the cavity photons.

Let us consider the same quantum-state diffusion unraveling as in the main text. For a time-independent operator $\hat{O}$, the stochastic evolution of its expectation is given by

$$\begin{aligned} \mathrm{d}\langle \hat{O} \rangle = \mathrm{i}\mathrm{d}t \Big\langle [\hat{H}, \hat{O}] \Big\rangle + \kappa \mathrm{d}t \Big\langle \hat{a}^\dagger \hat{O} \hat{a} - \frac{1}{2}\Big\{\hat{a}^\dagger \hat{a}, \hat{O}\Big\} \Big\rangle \\ + \sqrt{\kappa} \Big\{ \mathrm{d}Z^* \Big(\langle \hat{O} \hat{a} \rangle - \langle \hat{O} \rangle \langle \hat{a} \rangle \Big) + \\ \mathrm{d}Z \Big(\langle \hat{a}^\dagger \hat{O} \rangle - \langle \hat{a}^\dagger \rangle \langle \hat{O} \rangle \Big) \Big\} \,, \end{aligned} \tag{S18}$$

where the single-channel noise $\mathrm{d}Z$ satisfies $\mathrm{d}Z^2 = 0$ and $|\mathrm{d}Z|^2 = \mathrm{d}t$. This corresponds to monitoring the cavity output field with a heterodyne-detection scheme.

A Equations of motion

As our model preserves the translational symmetry of the state on the level of single trajectories, within the Gaussian approximation for both the cavity and the spins, we only need to keep track of the following quantities to fully specify the state:

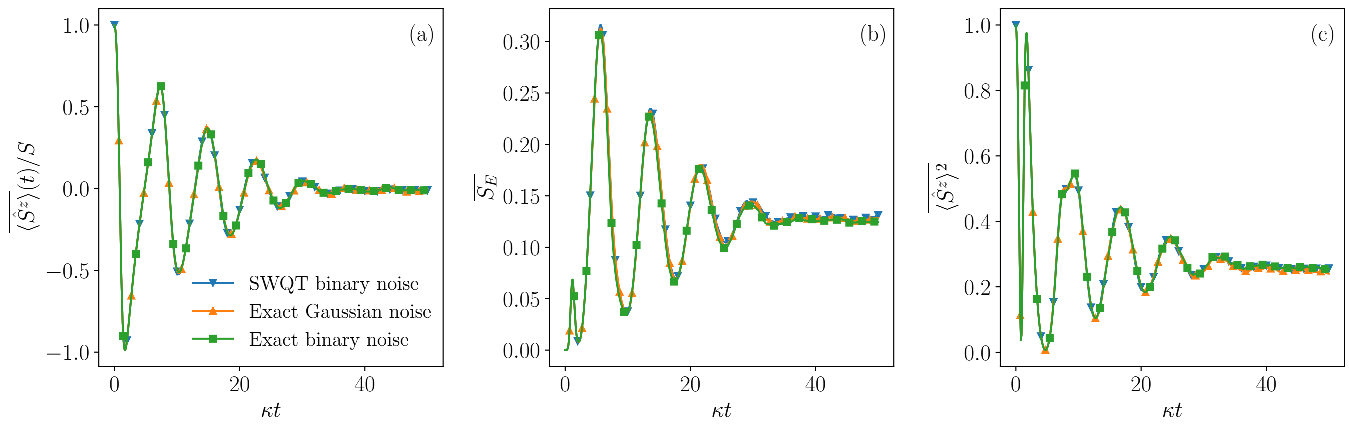

Supplementary Figure 9. Benchmark of the binary approximation of the Gaussian noise in the heterodyne-detection unraveling. We compare trajectory-averaged quantities obtained from different methods and different types of noise (see legend), namely 1) the spin-wave quantum trajectory (SWQT) method with binarized noise, 2) the exact solution of the stochastic master equation with Gaussian noise and 3) the exact solution but with binary noise replacing the gaussian noise. Panel (a) compares the results on a linear quantity $\overline{\langle \hat{S}^z \rangle}$. Panels (b) and (c) show the benchmark on two different nonlinear quantities, the half-system entanglement entropy $\overline{S_E}$ and the squared magnetization $\overline{\langle \hat{S}^z \rangle^2}$ respectively. Parameters: $\omega = 1.3\kappa$, $J = 0.1\kappa$, $\alpha = 0$ and $S = 64$.

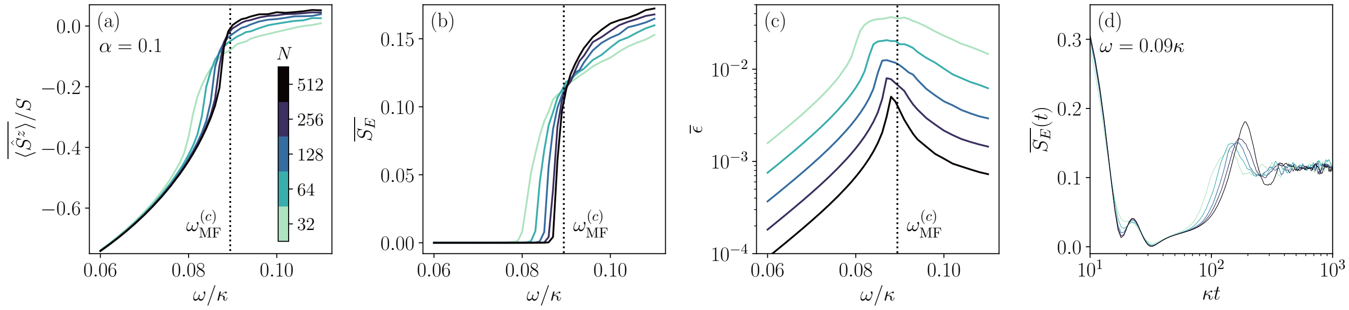

Supplementary Figure 10. Results for the power-law interacting Tavis-Cummings model with $\alpha = 0.1$, $J = 0.01\kappa$ and $\lambda = 0.2\kappa$ of different system sizes (colorbar shared across all panels). (a) Steady-state average z magnetization as a function of the drive ω. The vertical dotted line marks the critical point predicted by the mean-field theory $\omega_{\mathrm{MF}}^{(c)} \simeq 0.089\kappa$. (b) Steady-state of the trajectory-averaged entanglement entropy (between the spins and the cavity) as a function of ω. (c) Steady-state spin-wave density. (d) Dynamics of the trajectory-averaged spin-cavity entanglement entropy for a driving value $\omega = 0.09\kappa$, in linear-log scale.

- First moments: $\alpha \equiv \langle \hat{a} \rangle$, $\beta \equiv \langle \hat{b}_{n_0} \rangle$ (where n_0 is an arbitrary spin index, whose value has no importance due to the translational invariance of the spins.).
- Second moments:
  - Photon-photon correlations: $u^{(a)} \equiv \langle \hat{\delta}^{(a)} \hat{\delta}^{(a)} \rangle$, $v^{(a)} \equiv \langle \hat{\delta}^{(a)\dagger} \hat{\delta}^{(a)} \rangle$.
  - Photon-spin correlations: $u^{(ab)} \equiv \langle \hat{\delta}^{(a)} \hat{\delta}^{(b)}_{n_0} \rangle$, $v^{(ab)} \equiv \langle \hat{\delta}^{(a)\dagger} \hat{\delta}^{(b)}_{n_0} \rangle$.
  - Spin-spin correlations: $u^{(b)}_m \equiv \langle \hat{\delta}^{(b)}_{n_0} \hat{\delta}^{(b)}_{n_0+m} \rangle$, $v^{(b)}_m \equiv \langle \hat{\delta}^{(b)\dagger}_{n_0} \hat{\delta}^{(b)}_{n_0+m} \rangle$.

Here, we define $\hat{\delta}^{(a)} \equiv \hat{a} - \langle \hat{a} \rangle$ and $\hat{\delta}^{(b)}_i \equiv \hat{b}_i - \langle \hat{b}_i \rangle$, which are time-dependent operators. The spin correlators satisfy $u^{(b)}_m = u^{(b)}_{-m}$ and $v^{(b)}_m = v^{(b)}_{-m}$ due to spatial reflection symmetry in the considered model.

In the re-alignment step, as the rotation applies only to the spin operators, the cavity mode is not affected. Therefore, after obtaining the angular increments $\Delta\theta$ and $\Delta\phi$ from the incremented β using Eq. (28) in the main text, we have the following update rules,

$$\begin{aligned} \beta &\leftarrow 0\,, \\ u^{(ab)} &\leftarrow u^{(ab)} \mathrm{e}^{-\mathrm{i}\Delta\phi\cos\theta}\,, \\ v^{(ab)} &\leftarrow v^{(ab)} \mathrm{e}^{-\mathrm{i}\Delta\phi\cos\theta}\,, \\ u^{(b)}_m &\leftarrow u^{(b)}_m \mathrm{e}^{-2\mathrm{i}\Delta\phi\cos\theta}\,, \end{aligned} \tag{S19}$$

and the other correlators remain unchanged.

B Results on the driven-dissipative long-range Tavis-Cummings model

Note that in the regime where the cavity dissipation is much faster than any other time scale in the system, i.e. when $\omega, J, \lambda \ll \kappa$, the cavity mode can be adiabatically eliminated and this model reduces effectively to the long-range spin model with collective spin dissipation $\hat{S}^-$ as considered in the main text, since the lossy cavity acts as a Markovian reservoir for the spin system that quickly evacuates entropy into the environment via the spin-boson interaction. To illustrate the generalization of our spin-wave quantum trajectory method to spin-boson systems, we place ourselves close to this regime, such that results qualitatively reminiscent of those in the spin-only model as presented in the main text can be expected. We fix the parameters as $s = 1/2$, $\lambda = 0.2\kappa$, $J = 0.01\kappa$ and $\alpha = 0.1$, and consider a 1D spin chain in the cavity. The results are shown in Supplementary Fig. 10. The spin magnetization [panel (a)] displays similar behavior as compared to Fig. 3 (a) of the main text, where a continuous dissipative phase transition predicted by the mean-field theory emerges. This transition is also accompanied by an entanglement phase transition [panel (b)] for the entanglement between the spins and the cavity, from an unentangled phase to an entangled one as the driving increases. The spin-wave density [panel (c)] displays a maximum close to the critical point, while the peak value decreases with the system size N, which is a signature of the long-range nature of the system. Finally, the spin-cavity entanglement also exhibits fast saturation, as shown in panel (d) for the case close to criticality. These results suggest a possible implementation of the long-range spin model with collective dissipation (as considered in the main text) in atom-cavity platforms, and that the brute-force experimental detection of the entanglement phase transition can also be realized with mitigated post-selection overhead.

SUPPLEMENTARY REFERENCES

[1] H. M. Wiseman and G. J. Milburn, *Quantum measurement and control* (Cambridge university press, 2009).

[2] A. Serafini, F. Illuminati, and S. De Siena, Symplectic invariants, entropic measures and correlations of Gaussian states, J. Phys. B **37**, L21 (2003).

[3] K. Seetharam, A. Lerose, R. Fazio, and J. Marino, Dynamical scaling of correlations generated by short- and long-range dissipation, Phys. Rev. B **105**, 184305 (2022).
